# The impact of fluid balance on intracranial pressure in patients with traumatic brain injury

**DOI:** 10.1186/2197-425X-3-S1-A439

**Published:** 2015-10-01

**Authors:** E Moore, ER Saxby, J Wang, M Pilcher, D Bailey, S Heritier, O Huet, A Udy, DJ Cooper

**Affiliations:** Department of Epidemiology & Preventive Medicine, Monash University, Melbourne, Australia; The Alfred Hospital, Intensive Care Unit, Melbourne, Australia

## Introduction

Traumatic brain injury (TBI) is a huge public health problem leading to great personal suffering to victims and relatives and huge costs to society. Patients with TBI typically require substantial fluid replacement and resuscitation. However, evidence suggests that fluid accumulation, which could potentially increase cerebral swelling, may be associated with an increase in the incidence of elevated intracranial pressure (ICP) in patients with TBI^1^. We hypothesized that fluid balance (FB) after the first 48 hours following injury would be an independent predictor of ICP over the next five days.

## Objectives

To assess the relationship between FB and ICP in patients with severe TBI from day 3 to 7 after injury.

## Methods

We conducted a retrospective analysis of 100 adult trauma patients admitted to the ICU of a tertiary major trauma centre from Oct 2010 to Jun 2013 with head injury +/- multitrauma, who had an ICP monitor inserted for ≥ 7 days. FB for day 3 to 7 was calculated from the daily ICU charts (all fluid input minus all fluid output for this period). Mean ICP for day 3 to 7 was calculated from all end-hourly measurements taken on patients for this period. Further demographic, pre-hospital, clinical and biochemical data were collected from pathology, ICU and trauma registry databases. All repeated measures were averaged from day 3 to 7. We compared mean ICP for day 3 to 7 between patients with a positive versus negative FB for day 3 to 7. FB for day 3 to 7 and potential confounders were used in univariable and multivariable linear regression analyses with mean ICP for day 3 to 7 (Table 1).

**Table 1:** Linear regression: mean ICP and FB, day 3 to 7.

	Univariable		Multivariable	
Variable	β coefficient	p-value	β coefficient	p-value
**Fluid balance (L), day 3-7**	0.36 ± 0.09	< 0.001	0.17 ± 0.08	0.04
**Highest Na+, day 3-7**	0.25 ± 0.04	< 0.001	0.16 ± 0.04	0.001
**Mean temperature, day 3-7**	-1.12 ± 0.30	< 0.001	-0.56 ± 0.27	0.04
**Opioids (L), day 3-7**	1.43 ± 0.41	0.001	0.59 ± 0.77	0.44
**Midazolam (L), day 3-7**	1.70 ± 0.40	< 0.001	0.52 ± 0.79	0.51
**Male gender**	1.82 ± 0.89	0.04	1.72 ± 0.72	0.02
**Age (year)**	-.055 ± 0.02	0.02	-0.04 ± 0.02	0.05

## Results

Median age of the patients was 30 years, 83% were male and mean injury severity score (1998 version) was 37. Fifteen patients died in hospital, and median ICU length of stay was 16 days. Fifty three percent of patients had a positive FB and 47% had a negative FB for day 3 to 7. Patients with a positive versus negative FB for day 3 to 7 had a higher mean ICP (13.9 v 12.6 mmHg, p = 0.05).

After adjustment for confounders, a more positive FB for day 3 to 7 was significantly associated with an increase in mean ICP for day 3 to 7 (p = 0.04, R^2^ for model = 0.46).

## Conclusions

A more positive FB for day 3 to 7 post TBI was independently associated with an increased risk for a higher ICP. Further interventional studies aimed at testing the value of controlling FB in TBI patients after the 'critical' resuscitation period are warranted.
